# Psychopathic Personality Traits Scale (PPTS): Construct Validity of the Instrument in a Sample of U.S. Prisoners

**DOI:** 10.3389/fpsyg.2018.01596

**Published:** 2018-08-28

**Authors:** Daniel Boduszek, Agata Debowska, Nicole Sherretts, Dominic Willmott

**Affiliations:** ^1^Department of Psychology, University of Huddersfield, Huddersfield, United Kingdom; ^2^Katowice Faculty, SWPS University of Social Sciences and Humanities, Warsaw, Poland; ^3^Department of Psychology, University of Sheffield, Sheffield, United Kingdom

**Keywords:** psychopathy, Psychopathic Personality Traits Scale (PPTS), U.S. prisoners, multitrait-multimethod analysis, type of offences

## Abstract

The Psychopathic Personality Traits Scale (PPTS; Boduszek et al., 2016) is a personality-based psychopathy assessment tool consisting of four subscales: affective responsiveness, cognitive responsiveness, interpersonal manipulation, and egocentricity. Although the measure offers a promising alternative to other, more behaviorally weighted scales, to date the factor structure of the PPTS and differential predictive validity of its dimensions has only been tested in one study. Consequently, the objective of the present research was to assess construct validity, factor structure, and composite reliability of the PPTS within a sample of U.S. male and female incarcerated offenders (*N* = 772). Another goal was to test the predictive efficiency of the PPTS dimensions for different types of offences (serial killing, homicide, sex crimes, weapon-related crimes, domestic violence, white-collar crimes, property crimes, drug-related crimes), recidivism (i.e., number of incarcerations), time spent in prison, and gender. Dimensionality and construct validity of the PPTS was investigated using traditional CFA techniques, confirmatory bifactor analysis, and multitrait-multimethod modelling (MTMM). Seven alternative models of the PPTS were estimated in Mplus using WLSMV estimator. An MTMM model with four grouping factors (affective responsiveness, cognitive responsiveness, interpersonal manipulation, and egocentricity) while controlling for two method factors (knowledge/skills and attitudes/beliefs) offered the best representation of the data. Good composite reliability and differential predictive validity was reported. The PPTS can be reliably used among prisoners from the United States.

## Introduction

Psychopathy is a widely researched personality disorder (see Patrick, 2018 for a recent review of studies in the field). In spite of this, a unitary definition of psychopathy is missing, resulting in an ambiguous psychological construct (Ogloff, 2006; Buzina, 2012). Traditionally, researchers and clinicians have agreed that individuals with psychopathy are morally deprived, yet rational and able to differentiate between right and wrong (Arrigo and Shipley, 2001). In addition, early clinical observations demonstrated that highly psychopathic individuals can be abnormally impulsive and extremely violent (Ogloff, 2006). Cleckley (1941), based on psychiatric case studies, depicted psychopathic personalities as callous, grandiose, unreliable, dishonest, egocentric, as well as lacking empathy, regret, and remorse. Cleckley also argued for the existence of some adaptive traits among psychopathic individuals, such as resilience to anxiety, absence of irrational thinking, and rare instances of suicidality. In addition, Cleckley’s representation of psychopathy incorporated some behavioral characteristics, such as impulsivity and proneness to transgress social and legal norms. However, the latter set of traits was not central to psychopathy diagnosis in Cleckley’s writings.

Even though criminal tendencies featured in some early portrayals of psychopathic individuals (see Arrigo and Shipley, 2001 and Moreira et al., 2014 for a historical overview of psychopathy construct), observations upon which these were founded had been conducted in forensic and clinical settings, suggesting an overrepresentation of violent individuals in the samples used. The lack of early research with subclinical psychopaths, could have led to a distorted understanding of the essence of psychopathy, and, subsequently, an erroneous definition of the disorder. This conundrum appears to be reflected in some modern, widely used psychopathy assessment methods which tend to be weighted heavily toward behavioral expression of the disorder (for more details see Lilienfeld and Andrews, 1996; Boduszek and Debowska, 2016). A growing body of evidence shows that criminal/antisocial tendencies constitute a possible consequence rather than a fundamental part of psychopathy, indicating that such behaviors should be excluded from psychopathy assessment (Skeem and Cooke, 2010a,b; Boduszek et al., 2015; Cooke and Logan, 2015; Corrado et al., 2015; Boduszek and Debowska, 2016; Debowska et al., 2017).

Indeed, it has been established that psychopathic personalities can thrive in both criminal and noncriminal settings, including high risk sports, business, politics, the military, law enforcement, and firefighting (Babiak et al., 2010; Lilienfeld et al., 2012; Stevens et al., 2012; Hassall et al., 2015; Benning et al., 2018). Lilienfeld et al. (2012) conducted a study where 42 U.S. presidents were retrospectively assessed on psychopathy by historical experts. Preoffice psychopathy ratings were associated with various indicators of performance and results demonstrated that one of the psychopathic traits, fearless dominance, was related with better rated presidential performance, leadership, crisis management, and persuasiveness. To account for the fact that psychopathy is not found exclusively among criminals, Gao and Raine (2010) proposed a neurobiological theoretical model of successful and unsuccessful psychopathy. Based on a review of studies conducted with offending and nonoffending samples, the authors posited that successful psychopaths (i.e., those who evaded conviction for any criminal acts committed) have intact or enhanced executive functioning and cognitive empathy. This assertion is in line with Cleckley’s (1941) observation that some psychopathic individuals are characterized by superior intellectual abilities and, consequently, can be charming and highly manipulative. Further, according to Gao and Raine’s (2010) model, all psychopaths share similar deficits in emotional empathy, arousal, and emotion processing. Successful and unsuccessful psychopathy, therefore, appear to be characterized by different constellations of psychopathic traits, with successful psychopaths possessing more adaptive qualities than their unsuccessful counterparts (Lilienfeld et al., 2015).

In order to account for the variety of contexts in which psychopathic personalities can be found, Boduszek et al. (2016) proposed a pure personality-based psychopathy assessment without any behavioral indicators, the Psychopathic Personality Traits Scale (PPTS). The scale contains 20 items and has been intended for research purposes only. Grounded in Cleckley’s original conceptualization of psychopathy and recent empirical research, the PPTS consists of affective responsiveness, cognitive responsiveness, interpersonal manipulation, and egocentricity dimensions. More specifically, affective responsiveness refers to characteristics of low empathy and emotional shallowness. Cognitive responsiveness measures the ability to understand others’ emotional states, mentally represent another person’s emotional processes, and engage with others emotionally at a cognitive level. Interpersonal manipulation inquires into characteristics such as superficial charm, grandiosity, and deceitfulness. Finally, egocentricity is linked with incapacity for love other than self-love. In keeping with Gao and Raine’s (2010) model, Boduszek et al. (2016) theorized that cognitive responsiveness ratings will be inversely related to intelligence levels. More specifically, psychopathic individuals with superior intellectual abilities will be able to understand others’ emotional states. Highly psychopathic individuals with lower intelligence levels, on the other hand, will display deficits in cognitive responsiveness. According to the authors, affective responsiveness is not associated with intellectual abilities.

The PPTS has been validated among a large systematically selected prison sample from Poland (Boduszek et al., 2016). The researchers assessed seven alternative models of the PPTS, including a multitrait-multimethod (MTMM) model, also known as a correlated traits/correlated methods model, proposed by Campbell and Fiske (1959). The MTMM model consisting of four grouping factors (affective responsiveness, cognitive responsiveness, interpersonal manipulation, egocentricity) while controlling for two methods of measurement (a factor operationalized by items reflecting knowledge/skills and a factor operationalized by items reflecting attitudes/beliefs, independent of which grouping factor the items belong to) offered the best representation of the data. Noteworthy, the superiority of the MTMM model demonstrated the importance of controlling for measurement procedures not specific to the scale content in the assessment of psychopathy. More recently, Boduszek et al. (2017) performed a latent profile analysis using PPTS dimensions as indicators to determine psychopathy profiles among incarcerated offenders. Results revealed five distinct psychopathy groups, including a “high psychopathy group” (7.1% of the sample), “moderate psychopathy group” (10.8%), “high interpersonal manipulation group” (20.8%), “moderate affective/cognitive responsiveness group” (16.8%), and a “low psychopathy group” (44.6%). Boduszek et al. (2007) also reported that general violent offenders were most likely to belong in the “high psychopathy group,” whereas those convicted of property and white-collar offences were most likely to be the members of the “high interpersonal manipulation psychopathy group.”

Despite offering a promising alternative to other scales, to date the factor structure of the PPTS and differential predictive validity of its dimensions has only been tested among male inmates drawn from Polish prisons. As such, the scale’s psychometric properties warrant further assessment within more diverse populations. Indeed, prior research revealed differences in psychopathy scores and the expression of psychopathic traits between North American and European offending samples, which may be a function of differing socialization experiences (e.g., Cooke and Michie, 1999). In a more recent study, Verschuere et al. (2018) assessed the network structure of psychopathy as indexed using the Psychopathy Checklist – Revised (PCL-R; Hare, 2003) among U.S. and Dutch offender samples. Findings indicated that callous affect/lack of empathy was the most central traits in U.S. prisoners, whereas irresponsibility and parasitic lifestyle traits lay at the core of psychopathy in the Dutch sample. This disparity in the dominant characteristics points to the possible impact of culture on personality structures. Alternatively, the result may also be attributable to varying prison environments in different countries. Indeed, although personality traits have been traditionally conceptualized as relatively stable over time, recent research evidence suggests that life circumstances can stimulate changes in certain characteristics (see Bleidorn, 2012; Eriksson et al., 2017). The above findings combined demonstrate the necessity to validate psychopathy measures in samples from diverse backgrounds to verify their usefulness across settings.

### The Current Study

Thus, the objective of the present study was to verify whether the PPTS can be reliably used among English-speaking North American prisoners. Specifically, we wished to test construct validity, factor structure, and composite reliability of the PPTS within a sample of offenders from the U.S. prisons. In line with the supposition that criminal behavior may be an outcome of psychopathic personality traits, another goal was to test the utility of the PPTS dimensions for different types of offences (serial killing, homicide, sex crimes, weapon-related crimes, domestic violence, white-collar crimes, property crimes, drug-related crimes), recidivism (i.e., number of incarcerations), time spent in prison, and gender. Given the paucity of studies using the PPTS, we did not make any specific predictions as to the best model fit for the data or the nature of correlations between PPTS factors and external criteria.

## Materials and Methods

### Sample and Procedure

The data were collected in four prisons located in Pennsylvania (maximum security prison for males, *n* = 250; medium security for males, *n* = 186; maximum security for females, *n* = 223; and minimum security for females, *n* = 113). The project was approved by the Pennsylvania Department of Correction Ethics Committee.

Using convenience sampling, we approached 1000 inmates and 772 returned completed surveys (response rate = 77.20%). Printed self-reported anonymous surveys were delivered in envelopes by researchers to all selected prisons and opportunistically distributed among inmates. Given inmates’ standing as a vulnerable population and the potential that they may feel compelled to participate, it was made clear both in the consent form and verbally (by the prison personnel) that participation was voluntary. In addition, inmates were informed verbally that they should not participate in the study if they could not read in English, but that they did not have to inform data collectors of the specific reason for not participating in the study. Data collection occurred in inmates’ living units and was facilitated by one prison personnel on each block/wing. Surveys were collected by prison staff and returned to the research team. Due to the significant missing data for all variables (list-wise deletion method was used), 743 of inmates (418 males and 325 females) were included in the current analysis (age range from 20 to 77 years, *M* = 38.82, *SD* = 10.95, *Mdn* = 37, and Mode = 34).

Data on type of crime committed were collected using a self-reported checklist. Participants were asked to respond to the following categories: serial killing (more than two killings), homicide, sex crimes, crimes with weapon, domestic violence, white-collar crimes, property crimes, and drug-related crimes. Fifty-eight per cent of participants were convicted of more than one crime. Eighty-five (*n* = 85) participants indicated to have committed serial murders, 195 homicide, 125 weapon-related crimes, 344 property crimes (such as burglary and robbery), 200 drug-related offences, 116 sex offences, 19 domestic violence, and 62 white-collar crimes.

Three hundred and fifty-four (*n* = 354) participants were in prison for the first time, 160 for the second time, 84 for the third time, 52 for the fourth time, and 93 respondents were in prison five times or more (range from 1 to 20 times, *M* = 2.61, SD = 2.69, *Mdn* = 2, Mode = 1). Total time spent in prisons for the whole sample ranged from 1 to 792 months (*M* = 123.15, SD = 114.72, *Mdn* = 84, Mode = 60). We did not collect any additional socio-demographic data.

### Measure

Psychopathic Personality Traits Scale (PPTS; Boduszek et al., 2016) is a self-reported 20-item measure designed to assess psychopathic traits in forensic and nonforensic populations. The PPTS consists of four subscales: affective responsiveness (Factor 1; five items), cognitive responsiveness (Factor 2; five items), interpersonal manipulation (Factor 3; five items), and egocentricity (Factor 4; five items). All responses are indexed using *agree* (1) and *disagree* (0) format (i.e., a trait is either present or absent). Scores range from 0 to 20, with higher scores indicating increased levels of psychopathic traits. The affective responsiveness subscale assesses *lack* of empathy and emotional shallowness (higher scores suggest greater deficits in affective responsiveness). Cognitive responsiveness subscale refers to the ability to understand others’ emotional states, mentally represent another person’s emotional processes, and engage with others emotionally at a cognitive level (higher scores indicate greater deficits in cognitive responsiveness). The interpersonal manipulation subscale is used to measure characteristics such as superficial charm, grandiosity, and deceitfulness (higher scores indicate an increased ability to manipulate others). Egocentricity subscale measures an individual’s tendency to focus on one’s own interests, beliefs, and attitudes (higher scores suggest increased egocentricity). All items have been constructed to assess knowledge/skills or attitudes/beliefs as opposed to behaviors. Items 2, 6, 10, 13, 14, and 17 are reverse-scored.

### Data Analytic Plan

The dimensionality and construct validity of the PPTS was investigated through the application of traditional CFA techniques, confirmatory bifactor analysis (see Reise et al., 2010), and multitrait-multimethod modelling (MTMM). Seven alternative models of the PPTS latent structure were specified and tested using M*plus* version 7.4 (Muthén and Muthén, 1998/2015) with WLSMV estimation.

Model 1 is a one-factor solution where all PPTS items load on one latent factor of psychopathy. Model 2 is a correlated three-factor solution in which items 1, 2, 5, 6, 9, 10, 13, 14, 17, and 18 load on affective/cognitive responsiveness factor; items 3, 7, 11, 15, and 19 load on interpersonal manipulation factor; and items 4, 8, 12, 16, and 20 load on egocentricity factor. Model 3 is a bifactor solution with one general factor of psychopathy and three subordinate factors described in Model 2. Model 4 is an MTMM model composed of three grouping factors described in Model 5 and two correlated method factors: a factor operationalized by items reflecting knowledge/skills (M1; items 3, 7, 10, 11, 14, 15, 18, 19) and a factor operationalized by items reflecting attitudes/beliefs (M2; items 1, 2, 4, 5, 6, 8, 9, 12, 13, 16, 17, 20). Model 5 is a correlated four-factor solution where items 1, 5, 9, 13, and 17 load on affective responsiveness factor, items 2, 6, 10, 14, and 18 load on cognitive responsiveness factor, items 3, 7, 11, 15, and 19 load on interpersonal manipulation factor, items 4, 8, 12, 16, and 20 load on egocentricity factor. Model 6 is a bifactor solution with one general factor of psychopathy and four subordinate factors described in Model 5. Model 7 is an MTMM model including four grouping factors (as described in Model 5) and two correlated method factors (as described in Model 4).

The overall fit of each model and the relative fit between models were assessed using the following goodness-of-fit statistics: the χ2 statistic, the comparative fit index (CFI; Bentler, 1990), and the Tucker-Lewis index (TLI; Tucker and Lewis, 1973). For CFI and TLI, values above 0.90 and 0.95 indicate acceptable and good model fit, respectively (Bentler, 1990, 1995; Hu and Bentler, 1999). In addition, the root-mean-square error of approximation (RMSEA; Steiger and Lind, 1980) with 90% confidence interval is presented. Ideally, this index should be less than 0.05 to suggest good fit (Bentler, 1990; Hu and Bentler, 1999). Finally, the weighted root mean square residual (WRMR) was used to evaluate the alternative models, with the smallest value indicating the best-fitting model.

Differential predictive validity was assessed through the use of multiple regression (standardized regression coefficient = β with 95% CI was reported) for continuous outcome variables (time in prison, recidivism) and binary logistic regression [odds ratio (OR) with 95% CI was reported] for dichotomous outcome variables (gender and types of offences: serial killing, homicide, sex crimes, weapon-related crimes, domestic violence, white-collar crimes, robbery, drug-related crimes).

In contrast to previous research on validation of psychopathy construct which has typically assessed the internal consistency of items using alpha, the present study evaluated the internal reliability of the PPTS using composite reliability (for procedure see Raykov, 1997; for application in psychopathy research see Boduszek et al., 2015). Cronbach’s coefficient α assumes that all scale items have equal loadings on a single scale factor. If this assumption is not met, scale reliability is likely to be underestimated. Additionally, the underestimation of reliability by alpha is greater for scales with a small number of items (Yang and Green, 2011). Composite reliability is calculated based on standardized regression weights and does not assume that all items have equal loadings on a single factor (Raykov, 1997). Values greater than 0.60 are generally considered acceptable (Diamantopoulos and Siguaw, 2000).

## Results

### Descriptive Statistics

Descriptive statistics for the four PPTS factors [affective responsiveness (AR), cognitive responsiveness (CR), interpersonal manipulation (IPM), and egocentricity (EGO)] are presented in **Table 1** below.

**Table 1:** Descriptive statistics for PPTS factors.

Variables	*M*	*SD*	Mdn	Min	Max	Skewness	Kurtosis
Affective responsiveness	1.01	1.26	1	0	5	1.24	0.75
Cognitive responsiveness	1.22	1.16	1	0	5	0.82	0.10
Interpersonal manipulation	1.66	1.57	1	0	5	0.59	0.83
Egocentricity	1.93	1.22	2	0	5	0.27	0.50


*SD, standard deviation*.

### Confirmatory Factors Analyses Results and Correlations Between PPTS Dimensions

**FIGURE 1:**
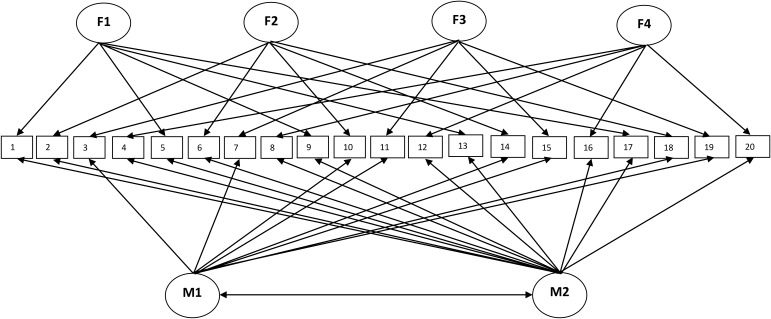
MTMM model of the PPTS. F1, affective responsiveness; F2, cognitive responsiveness; F3, interpersonal manipulation; F4, egocentricity; M1, knowledge/skills; and M2, attitudes/beliefs.

Fit indices for the seven alternative models of the PPTS are presented in **Table 2**. Models 1 (one factor), 2 (three factors), 3 (bifactor with three grouping factors), 5 (four factors), and 6 (bifactor with four grouping factors) were rejected based on the CFI and TLI (values below 0.90) and RMSEA (values above 0.05). Models 4 (MTMM with three grouping factors and two method factors) and 7 (MTMM with four grouping factors and two method factors) are acceptable solutions, with Model 7 providing the best fit to the data [CFI = 0.95, TLI = 0.93, RMSEA = 0.040 (90% CI = 0.034/0.046), WRMR = 1.07]. A visual representation of Model 7 is provided in **Figure 1**.

**Table 2:** Fit indices for seven alternative models of the PPTS.

Models	χ*^2^*	*Df*	CFI	TLI	RMSEA (90% CI)	WRMR
(1) One Factor	1202.99^∗∗∗^	170	0.69	0.66	0.089 (0.084/0.094)	2.41
(2) Three Factors	859.03^∗∗∗^	167	0.80	0.77	0.073 (0.069/0.078)	2.02
(3) Bifactor with 3 grouping factors	531.76^∗∗∗^	150	0.89	0.86	0.057 (0.052/0.063)	1.47
(4) MTMM (3 factors with 2 method factors)	398.67^∗∗∗^	146	0.93	0.90	0.047 (0.042/0.053)	1.20
(5) Four Factors	833.04^∗∗∗^	164	0.80	0.77	0.073 (0.068/0.078)	1.96
(6) Bifactor with 4 grouping factors	577.59^∗∗∗^	150	0.87	0.84	0.061 (0.056/0.066)	1.58
(7) MTMM (4 factors with 2 method factors)	322.14^∗∗∗^	143	0.95	0.93	0.040 (0.034/0.046)	1.07


*^∗∗∗^indicates χ*^*2*^* is statistically significant (*p* < 0.001). χ^*2*^, chi square goodness of fit statistic; *df*, degrees of freedom; CFI, Comparative Fit Index; TLI, Tucker Lewis Index; RMSEA, Root-Mean-Square Error of Approximation; CI, confidence interval; WRMR, Weighted Root Mean Square Residual.*

The adequacy of Model 7 can also be determined based on parameter estimates. As demonstrated in **Table 3**, all items showed statistically significant factor loadings. PPTS items loaded more strongly on grouping factors and less strongly on method factors, indicating the supremacy of the four grouping factors over the method factors in the conceptualization of the factor structure of the PPTS, as well as its related scoring scheme. These results reveal that the PPTS consist of four grouping factors (AR, CR, IPM, EGO) while controlling for the method of measurement (knowledge/skills and attitudes/beliefs).

**Table 3:** Standardized factor loadings for the four psychopathy factors (AR, affective responsiveness; CR, cognitive responsiveness; IPM, interpersonal manipulation; EGO, egocentricity) and two method factors (Method 1, knowledge/skills; Method 2, attitudes/beliefs) of the PPTS.

Original Item Numbers	Method 1	Method 2	AR	CR	IPM	EGO
(1) I don’t care if I upset someone to get what I want.		0.49^∗∗∗^	0.57^∗∗∗^			
(2) Before criticizing somebody, I try to imagine and understand how it would make them feel.		0.31^∗∗∗^		0.62^∗∗∗^		
(3) I know how to make another person feel guilty.	0.45^∗∗∗^				0.66^∗∗∗^	
(4) I tend to focus on my own thoughts and ideas rather than on what others might be thinking.		0.16^∗^				0.53^∗∗∗^
(5) What other people feel doesn’t concern me.		0.22^∗∗^	0.70^∗∗∗^			
(6) I always try to consider the other person’s feelings before I do something.		0.37^∗∗∗^		0.54^∗∗∗^		
(7) I know how to pay someone compliments to get something out of them.	0.57^∗∗∗^				0.69^∗∗∗^	
(8) I don’t usually appreciate the other person’s viewpoint if I don’t agree with it.		0.34^∗∗∗^				0.46^∗∗∗^
(9) Seeing people cry doesn’t really upset me.		0.33^∗∗∗^	0.72^∗∗∗^			
(10) I am good at predicting how someone will feel.	0.26^∗∗^			0.63^∗∗∗^		
(11) I know how to simulate emotions like pain and hurt to make others feel sorry for me.	0.53^∗∗∗^				0.72^∗∗∗^	
(12) In general, I’m only willing to help other people if doing so will benefit me as well.		0.46^∗∗∗^				0.47^∗∗∗^
(13) I tend to get emotionally involved with a friend’s problems.		0.10^∗^	0.53^∗∗∗^			
(14) I’m quick to spot when someone is feeling awkward or uncomfortable.	0.17^∗^			0.71^∗∗∗^		
(15) I sometimes provoke people on purpose to see their reaction.	0.28^∗∗∗^				0.48^∗∗∗^	
(16) I believe in the motto: “I’ll scratch your back, if you scratch mine”.		0.20^∗∗^				0.48^∗∗∗^
(17) I get filled with sorrow when people talk about the death of their loved ones.		0.31^∗∗∗^	0.65^∗∗∗^			
(18) I find it difficult to understand what other people feel.	0.14^∗∗^			0.44^∗∗∗^		
(19) I sometimes tell people what they want to hear to get what I want from them.	0.63^∗∗∗^				0.49^∗∗∗^	
(20) It’s natural for human behavior to be motivated by self-interest.		0.16^∗^				0.49^∗∗∗^


*Items 2, 6, 10, 13, 14, and 17 are reverse-scored. Factor loadings are statistically significant at ^∗^*p*< 0.05; ^∗∗^*p* < 0.01; ^∗∗∗^*p* < 0.001.*

**Table 4** displays correlations between all latent factors. These correlations ranged between weak to moderate. The lowest correlation was reported between IPM and CR (*r* = 0.10) as well as EGO and CR (*r* = 0.10). The highest correlation was found between the two method factors (*r* = 0.46).

**Table 4:** Associations between the PPTS factors.

Factor	AR	CR	IPM	EGO	M1	M2
Affective responsiveness (AR)	1					
Cognitive responsiveness (CR)	0.32^∗∗∗^	1				
Interpersonal manipulation (IPM)	0.20^∗∗∗^	0.10^∗∗∗^	1			
Egocentricity (Ego)	0.26^∗∗∗^	0.10^∗∗∗^	0.33^∗∗∗^	1		
M1 (knowledge/skills)	n/a	n/a	n/a	n/a	1	
M2 (attitudes/beliefs)	n/a	n/a	n/a	n/a	0.46^∗∗∗^	1


*^∗∗∗^*p*< 0.001*.

### Associations Between PPTS Factors and External Criteria

**Table 5** presents the outcome of multiple regression (time in prison, recidivism) and multiple logistic regression (gender, serial killing, homicide, sex crimes, weapon-related crimes, domestic violence, white-collar crimes, property crimes, drug-related crimes) analyses. Based on the statistics provided, time in prison forms a significant positive correlation with CR, whereas recidivism correlates positively with IPM. Females score significantly lower than males on AR. As for the different types of offences, AR associated positively with white-collar crimes, CR with serial killing, homicide, weapon-related crimes, and robbery. IPM correlated positively with white-collar crimes, robbery, drug-related crimes, and negatively with homicide. Lastly, EGO correlated positively with domestic violence.

**Table 5:** Associations between the four PPTS factors and gender [Female = 1 (*n* = 325), Male = 0 (*n* = 418)], Total time spent in prisons (time in prison), number of incarcerations (recidivism), serial killing, homicide, sex crime, weapon-related crime (weapon), domestic violence (domestic), white-collar crime, property crime, and drug-related crimes (drugs).

	Gender OR (95% CI)	Time in prison β (95% CI)	Recidivism β (95% CI)	Serial killing OR (95% CI)	Homicide OR (95% CI)	Sex crime OR (95% CI)	Weapon OR (95% CI)	Domestic OR (95% CI)	White-collar OR (95% CI)	Property OR (95% CI)	Drugs OR (95%CI)
AR	0.84^∗∗^ (0.73/0.96)	0.01 (-0.07/0.09)	0.06 (-0.02/0.14)	1.03 (0.83/1.27)	1.01 (0.87/1.17)	0.92 (0.77/1.12)	1.13 (0.96/1.32)	1.01 (0.70/1.44)	1.24^∗^ (1.01/1.52)	0.99 (0.85/1.16)	0.96 (0.83/1.11)
CR	0.96 (0.84/1.10)	0.08^∗^ (0.00/0.15)	0.01 (-0.07/0.09)	1.32^∗∗^ (1.07/1.62)	1.21^∗^ (1.04/1.41)	0.99 (0.82/1.20)	1.33^∗∗∗^ (1.12/1.58)	1.08 (0.71/1.64)	0.91 (0.71/1.17)	1.16^∗^ (1.01/1.37)	0.96 (0.82/1.12)
IPM	0.99 (0.89/1.096)	-0.05 (-0.13/.03)	0.10^∗^ (0.02/0.17)	0.91 (0.76/1.08)	0.88^∗^ (0.78/0.99)	0.93 (0.80/1.08)	1.10 (0.96/1.27)	1.07 (0.79/1.45)	1.25^∗∗^ (1.04/1.49)	1.13^∗^ (1.00/1.28)	1.21^∗∗∗^ (1.08/1.36)
EGO	0.99 (0.87/1.14)	0.02 (-0.06/0.10)	0.07 (-0.01/0.15)	0.91 (0.73/1.13)	0.95 (0.81/1.10)	1.01 (0.84/1.21)	0.98 (0.81/1.17)	1.90^∗∗∗^ (1.24/2.90)	1.02 (0.81/1.30)	1.04 (0.89/1.23)	1.07 (0.93/1.25)


*Results from multiple logistic regression (OR with 95% CI) and multiple regression analyses (β with 95% CI). ^∗^*p* < 0.05, ^∗∗^*p*< 0.01, ^∗∗∗^*p*< 0.001.*

### Composite Reliability Results

Composite reliability was calculated to determine the internal reliability of the PPTS factors. All four psychopathy factors (AR = 0.77, CR = 0.73, IPM = 0.75, and EGO = 0.61) demonstrate adequate to good internal reliability.

## Discussion

The PPTS (Boduszek et al., 2016) constitutes a promising alternative to other scales indexing criminal/antisocial behavior, but its validity and predictive efficiency has only been tested in one, exclusively male, Polish offender sample. Accordingly, the first aim of this study was to validate the PPTS among a mixed-gender sample of prisoners drawn from U.S. prisons, using confirmatory factor techniques. The second aim was to test the predictive utility of the PPTS dimensions for different types of offences (serial killing, homicide, sex crimes, weapon-related crimes, domestic violence, white-collar crimes, property crimes, drug-related crimes), recidivism (i.e., number of incarcerations), and time spent in prison. We also examined associations between the PPTS facets and gender.

In the first validation of the PPTS among Polish prisoners by Boduszek et al. (2016), a multitrait-multimethod (MTMM) model including four grouping factors (affective responsiveness, cognitive responsiveness, interpersonal manipulation, and egocentricity) and two correlated method factors (knowledge/skills and attitudes/beliefs) offered the best fit for the data. Since factor loadings were stronger for the grouping factors compared with the method factors, it was proposed that the grouping factors should provide the basis for creating the PPTS subscales (in line with Reise et al., 2010). In the current sample, we tested seven theoretically derived models of the PPTS. Two MTMM models proposed an adequate fit to the data, with the MTMM model consisting of four grouping factors and two correlated method factors providing the best representation for the data. Grouping factors recorded higher factor loadings than method factors, suggesting that the PPTS should be conceptualized to consist of four subscales (affective responsiveness, cognitive responsiveness, interpersonal manipulation, egocentricity) when utilized among U.S. incarcerated offenders. Therefore, the current findings are fully reflective of those reported in the initial PPTS validation and indicate that the measure can be used in the same way among Polish and U.S. prisoners. In light of prior research suggesting that psychopathy may differ across cultures (e.g., Cooke and Michie, 1999; Verschuere et al., 2018), this in an interesting finding. More specifically, the current study provides preliminary evidence that the PPTS factor structure remains stable across samples drawn from two culturally diverse prison settings and hence the PPTS can be used in the same way regardless of prison context. This may be due to the exclusion of criminal/antisocial traits and items referring to behavior in general, which can be environment specific. We hypothesize that the constellations of psychopathic traits may vary for different offending populations, but this assertion should be tested in future research using person-centered analytic techniques.

Further, we sought to determine whether scores on individual PPTS factors correlate with recidivism, time spent in prison, and different types of offences. We found that interpersonal manipulation was positively related with recidivism, white-collar crimes, property crimes, and drug-related offences. One possible explanation of this finding is that people who commit crimes associated with financial gain, and in particular those who do it repeatedly, possess (or develop in the process of their criminal careers) strong interpersonal manipulation skills, which may be crucial in deceiving others to one’s own benefit. This is in line with Boduszek et al.’s (2017) research showing that high scores on interpersonal manipulation combined with low scores on the remaining factors were associated with property and white-collar offending.

Next, we found heightened egocentricity ratings among domestic violence perpetrators. This suggests that it may be difficult for such individuals to assume or understand their partner’s perspective, resulting in violence if, for example, the partner is unwilling to share and/or agree with the perpetrator’s point of view. This finding and the explanation offered are in keeping with Schweinle et al. (2010) language analysis of maritally aggressive men. More specifically, the researchers demonstrated that violent husbands use egocentric words (such as first-person pronouns) in describing their marriages.

Further, increased deficits in cognitive responsiveness were recorded for offenders convicted of homicide, serial killing, weapon-related offences, and property crimes. It therefore appears that inability to engage with others emotionally at a cognitive level, may lead to criminal behavior in general, rather than a specific form of criminal conduct. If future research with more diverse samples substantiates the above supposition, techniques focusing on sensitization to others’ emotional states should be contained within prevention and intervention programs. Given increased cognitive malleability in youngsters (Birch et al., 2017), it is theorized that such tactics would be particularly effective in curtailing youth offending. Deficits in cognitive responsiveness were also positively correlated with total time spent in prison. Although we could not test this in the current study, it appears that this association can be moderated by intelligence levels. In agreement with Gao and Raine’s (2010) model of successful and unsuccessful psychopathy, individuals with increased deficits in cognitive responsiveness and lowered levels of intelligence are more likely to commit more crimes and be convicted for them, and in consequence spend more time in prison.

Decreased affective responsiveness was associated with white-collar offending, i.e., a type of nonviolent crime. Although the result was somewhat unexpected, prior research inquiring into the role of callous-unemotional (CU) traits (e.g., lack of empathy and remorse, shallow affect) in criminal offending among young men, indicated that CU traits were predictive of theft (such as burglary, fraud, forgery) in Caucasian but not African American men. This may mean that CU traits form weaker associations with criminality among minorities (Kahn et al., 2013). Future research using the PPTS should account for racial/ethnic groups to determine whether a similar effect exists for the affective responsiveness dimension. Finally, congruent with prior suggestions that women are more emotionally empathetic than men (e.g., Mestre et al., 2009), female offenders in the current sample were found to be le ss likely to express deficits in affective responsiveness. No further gender differences in scores on PPTS dimensions were detected.

This study has several methodological weaknesses. First, the sample was limited to English-speaking prisoners which might limit generalization to other U.S. prisoners whose command of English was not sufficient to participate in the study. We did not collect information on how many individuals did not take part due to the language barrier. In addition, the data presented here were cross-sectional and thus conclusions about temporal and causal relationships between PPTS factors and external criteria cannot be derived. Next, we asked participants to self-report crimes that they had committed. Future studies should seek to validate prisoners’ responses against official records. Further, although the sample consisted of male and female prisoners, these samples were not big enough to allow for testing for factorial invariance of the PPTS. Future work is needed to address all above-cited limitations. We particularly encourage testing for psychopathy among youth offenders before and after exposure to intervention programs, especially those involving perspective taking techniques, to determine whether such strategies can lead to the alleviation of certain psychopathic traits. Future research should also control for intelligence levels, to determine whether deficits in cognitive responsiveness are more pronounced in individuals with decreased executive functioning. Studies are also needed to verify whether the PPTS can be reliably used with nonoffending populations.

To summarize, PPTS scores among U.S. prisoners are best captured by an MTMM model consisting of four grouping factors (affective responsiveness, cognitive responsiveness, interpersonal manipulation, egocentricity) and two method factors (knowledge/skills and attitudes/beliefs). PPTS dimensions formed differential associations with external criteria, including those referring to different types of offending. Future work can contribute to further development of the new theoretical approach to defining psychopathy as grasped by the PPTS, as well as more reliable psychopathy assessment.

## Ethics Statement

This study was carried out in accordance with the recommendations of American Psychological Association Guidelines. The protocol was approved by the Pennsylvania Department of Correction Ethics Committee. All subjects gave written informed consent in accordance with the Declaration of Helsinki.

## Author Contributions

DB designed the study, analyzed the data, and wrote the article. AD designed the study and wrote the article. NS did data collection, data entry, and proofreading of the article. DW wrote the article.

## Conflict of Interest Statement

The authors declare that the research was conducted in the absence of any commercial or financial relationships that could be construed as a potential conflict of interest.

## References

[B1] Arrigo B. A. Shipley S. (2001). The confusion over psychopathy (I): historical considerations. *Int. J. Offender Ther. Comp. Criminol.* 45 325–344. 10.1177/0306624X01453005

[B2] Babiak P. Neumann C. S. Hare R. D. (2010). Corporate psychopathy: talking the walk. *Behav. Sci. Law* 28 174–193. 10.1002/bsl.925 20422644

[B3] Benning S. D. Venables N. C. Hall J. R. (2018). “Successful psychopathy”, in *Handbook of Psychopathy*, 2nd Edn, ed. Patrick C. J. (New York, NY: Guilford Press), 585–609.

[B4] Bentler P. M. (1990). Comparative fit indices in structural models. *Psychol. Bull.* 217 238–246. 10.1037/0033-2909.107.2.238 2320703

[B5] Bentler P. M. (1995). *EQS Structural Equations Program Manual.* Encino, CA: Multivariate Software.

[B6] Birch S. A. J. Li V. Haddock S. E. Ghrear S. E. Brosseau-Liard P. Baimel A. (2017). “Perspectives on perspective taking: how children think about the minds of others,” in *Advances in Child Development and Behavior*, Vol. 52 ed. Benson J. B. (Cambridge, MA: Elsevier), 185–226. 10.1016/bs.acdb.2016.10.005 28215285

[B7] Bleidorn W. (2012). Hitting the road to adulthood: short-term personality development during a major life transition. *Pers. Soc. Psychol. Bull.* 38 1594–1608. 10.1177/0146167212456707 22894876

[B8] Boduszek D. Debowska A. (2016). Critical evaluation of psychopathy measurement (PCL-R and SRP-III/SF) and recommendations for future research. *J. Crim. Justice* 44 1–12. 10.1016/j.jcrimjus.2015.11.004

[B9] Boduszek D. Debowska A. Dhingra K. DeLisi M. (2016). Introduction and validation of Psychopathic Personality Traits Scale (PPTS) in a large prison sample. *J. Crim. Justice* 46 9–17. 10.1016/j.jcrimjus.2016.02.004

[B10] Boduszek D. Debowska A. Willmott A. (2017). Latent profile analysis of psychopathic traits among homicide, general violent, property and white-collar offenders. *J. Crim. Justice.* 51 17–23. 10.1016/j.jcrimjus.2017.06.001

[B11] Boduszek D. Dhingra K. Hyland P. Debowska A. (2015). A bifactorial solution to the psychopathy checklist: screening version in a sample of civil psychiatric patients. *Crim. Behav. Ment. Health* 26 174–185. 10.1002/cbm.1956 25832999

[B12] Buzina N. (2012). Psychopathy–historical controversies and new diagnostic approach. *Psychiatr. Danub.* 24 134–142. 22706411

[B13] Campbell D. T. Fiske D. W. (1959). Convergent and discriminant validation by the multitrait-multimethod matrix. *Psychol. Bull.* 56 81–105. 10.1037/h0046016 13634291

[B14] Cleckley H. (1941). *The Mask of Sanity*, 1st Edn. St. Louis, MO: C.V. Mosby.

[B15] Cooke D. J. Logan C. (2015). Capturing clinical complexity: towards a personality-oriented measure of psychopathy. *J. Crim. Justice* 43 262–273. 10.1016/j.jcrimjus.2015.04.004

[B16] Cooke D. J. Michie C. (1999). Psychopathy across cultures: North America and Scotland compared. *J. Abnorm. Psychol.* 108 58–68. 10.1037/0021-843X.108.1.58 10066993

[B17] Corrado R. R. DeLisi M. Hart S. D. McCuish E. C. (2015). Can the causal mechanisms underlying chronic, serious, and violent offending trajectories be elucidated using the psychopathy construct? *J. Crim. Justice* 43 251–261. 10.1016/j.jcrimjus.2015.04.006

[B18] Debowska A. Boduszek D. Dhingra K. Sherretts N. Willmott D. DeLisi M. (2017). Can we use Hare’s psychopathy model within forensic and non-forensic populations? An empirical investigation. *Deviant Behav.* 39 224–242. 10.1080/01639625.2016.1266887

[B19] Diamantopoulos A. Siguaw J. A. (2000). *Introducing LISREL.* London: Sage Publications.

[B20] Eriksson T. G. Masche-No J. G. Dåderman A. M. (2017). Personality traits of prisoners as compared to general populations: signs of adjustment to the situation? *Pers. Individ. Dif.* 107 237–245. 10.1016/j.paid.2016.11.030

[B21] Gao Y. Raine A. (2010). Successful and unsuccessful psychopaths: a neurobiological model. *Behav. Sci. Law* 28 194–210. 10.1002/bsl.924 20422645

[B22] Hare R. D. (2003). *The Hare Psychopathy Checklist—Revised. 2.* Toronto, ON: Multi-Health Systems.

[B23] Hassall J. Boduszek D. Dhingra K. (2015). Psychopathic traits of business and psychology students and their relationship to academic success. *Pers. Individ. Dif.* 82 227–231. 10.1016/j.paid.2015.03.017

[B24] Hu L. Bentler P. M. (1999). Cutoff criteria for fit indexes in covariance structure analysis: conventional criteria versus new alternatives. *Struct. Equat. Model.* 6 1–55. 10.1080/10705519909540118

[B25] Kahn R. E. Byrd A. L. Pardini D. A. (2013). Callous-unemotional traits robustly predict future criminal offending in young men. *Law Hum. Behav.* 37 87–97. 10.1037/b0000003 22731505 PMC3822438

[B26] Lilienfeld S. O. Andrews B. P. (1996). Development and preliminary validation of a self-report measure of psychopathic personality traits in noncriminal population. *J. Pers. Assess.* 66 488–524. 10.1207/s15327752jpa6603_3 8667144

[B27] Lilienfeld S. O. Waldman I. D. Landfield K. Watts A. L. Rubenzer S. Faschingbauer T. R. (2012). Fearless dominance and the US presidency: implications of psychopathic personality traits for successful and unsuccessful political leadership. *J. Pers. Soc. Psychol.* 103 489–505. 10.1037/a0029392 22823288

[B28] Lilienfeld S. O. Watts A. L. Smith S. F. (2015). Successful psychopathy: a scientific status report. *Curr. Dir. Psychol. Sci.* 24 298–303. 10.1177/0963721415580297

[B29] Mestre M. V. Samper P. Frías M. D. Tur A. M. (2009). Are women more empathetic than men? A longitudinal study in adolescence. *Span. J. Psychol.* 12 76–83. 10.1017/S1138741600001499 19476221

[B30] Moreira D. Almeida F. Pinto M. Fávero M. (2014). Psychopathy: a comprehensive review of its assessment and intervention. *Aggress. Violent Behav.* 19 191–195. 10.1016/j.avb.2014.04.008

[B31] Muthén L. K. Muthén B. O. (1998–2015). *Mplus User’s Guide*, 7th Edn. Los Angeles, CA: Muthén & Muthén.

[B32] Ogloff J. R. P. (2006). Psychopathy/antisocial personality disorder. *Aust. N. Z. J. Psychiatry* 40 519–528. 10.1080/j.1440-1614.2006.01834.x 16756576

[B33] Patrick C. J. (ed.) (2018). *Handbook of Psychopathy*, 2nd Edn. New York, NY: Guilford Press.

[B34] Raykov T. (1997). Estimation of composite reliability for congeneric measures. *Appl. Psychol. Meas.* 21 173–184. 10.1177/01466216970212006

[B35] Reise S. P. Moore T. M. Haviland M. G. (2010). Bifactor models and rotations: exploring the extent to which multidimensional data yield univocal scale scores. *J. Pers. Assess.* 92 544–559. 10.1080/00223891.2010.496477 20954056 PMC2981404

[B36] Schweinle W. Ickes W. Rollings K. Jacquot C. (2010). Maritally aggressive men: angry, egocentric, impulsive and/or biased. *J. Lang. Soc. Psychol.* 29 399–424. 10.1177/0261927X10377988

[B37] Skeem J. L. Cooke D. J. (2010a). Is criminal behavior a central component of psychopathy? Conceptual directions for resolving the debate. *Psychol. Assess.* 22 433–445. 10.1037/a0008512 20528069

[B38] Skeem J. L. Cooke D. J. (2010b). One measure does not a construct make: directions toward reinvigorating psychopathy research—reply to hare and neumann (2010). *Psychol. Assess.* 22 455–459. 10.1037/a0014862 20528071

[B39] Steiger J. H. Lind J. C. (1980). Statistically based tests for the number of common factors. *Paper Presented at the Annual Meeting of the Psychometric Society*, Vol. 758 Iowa City, IA.

[B40] Stevens G. W. Deuling J. K. Armenakis A. A. (2012). Successful psychopaths: are they unethical decision-makers and why? *J. Bus. Ethics* 105 139–149. 10.1007/s10551-011-0963-1

[B41] Tucker L. R. Lewis C. (1973). A reliability coefficient for maximum likelihood factor analysis. *Psychometrika* 38 1–10. 10.1007/BF02291170

[B42] Verschuere B. van Ghesel Grothe S. Waldorp L. Watts A. L. Lilienfeld S. O. Edens J. F. (2018). What features of psychopathy might be central? A network analysis of the psychopathy checklist-revised (PCL-R) in three large samples. *J. Abnorm. Psychol.* 127 51–65. 10.1037/abn0000315 29172600

[B43] Yang Y. Green S. B. (2011). Coefficient alpha: a reliability coefficient for the 21st century? *J. Psychoeduc. Assess.* 29 377–392. 10.1177/0734282911406668 29025432

